# TOPical Imiquimod treatment of residual or recurrent cervical intraepithelial neoplasia (TOPIC-2 trial): a study protocol for a randomized controlled trial

**DOI:** 10.1186/s12885-018-4510-7

**Published:** 2018-06-15

**Authors:** A. J. M. van de Sande, M. M. Koeneman, C. G. Gerestein, A. J. Kruse, F. J. van Kemenade, H. J. van Beekhuizen

**Affiliations:** 1 Department of Obstetrics and Gynecology, Erasmus Medical Center Cancer Institute, Post box 2040, 3000 CA Rotterdam, The Netherlands; 2 Department of Obstetrics and Gynecology, Meander Medical Center, Amersfoort, The Netherlands; 3 Department of Obstetrics and Gynecology, Maastricht University Medical Center, Maastricht, The Netherlands

**Keywords:** Cervical intraepithelial neoplasia, Imiquimod, Human papillomavirus, Quality of life, Dysplasia cervix, LLETZ, Transformation zone

## Abstract

**Background:**

Cervical dysplasia (cervical intraepithelial neoplasia (CIN)) is caused by Human Papillomavirus (HPV) and is most common in women of reproductive age. Current treatment of moderate to severe CIN is surgical. This procedure has potential complications, such as haemorrhage, infection and preterm birth in subsequent pregnancies. Moreover, 15% of women treated for high grade CIN develop residual/recurrent CIN or cervical cancer after surgical excision. Finally, 75–100% of patients with a residual and recurrent CIN 2–3 lesion are still HPV positive. They could possibly benefit from an alternative medical treatment, which aims to eliminate HPV.

The primary study objective is to evaluate the effectivity of imiquimod 5% cream compared to treatment with Large Loop Excision of the Transformation Zone (LLETZ) for recurrent/residual CIN.

**Methods/design:**

This study is a multicentre, non-inferiority randomized single blinded study. The study population consists of female patients with histological proven residual/recurrent CIN after previous surgical treatment. Four hundred thirty-three patients will be included in the Netherlands. The first 35 patients will be included in a pilot study to prove non-futility.

Included patients will be randomized to receive either 5% imiquimod cream or LLETZ treatment. Imiquimod will be inserted three times a week intravaginally for a period of 16 weeks using a vaginal applicator. Ten weeks after the end of imiquimod treatment a biopsy will be taken for treatment response. In case of progressive or stable disease a LLETZ will be performed. At 12 and 24 months after the start of treatment cytology will be taken for follow up. The LLETZ group will be treated according to the current guidelines. Throughout the study, HPV typing and quality of life will be tested.

**Discussion:**

Repeated LLETZ in women with residual/recurrent CIN lesions has complications. We would like to possibly offer alternative treatment in a selected group to avoid these risks. Moreover, we monitor treatment efficacy, side effects and long-term recurrence rates.

**Trial registration:**

Medical Ethical Committee approval number: NL 53792.078.15. Affiliation: Erasmus Medical Center.

Registration number ClinicalTrials.gov: NCT02669459, date of registration: 27th January 2016.

## Background

Cervical dysplasia is caused by Human Papillomavirus (HPV) and is most common in women of reproductive age. Cervical dysplasia is known to be a precancerous stage of cervical cancer, the fourth most common type of cancer worldwide in women [1]. Treatment of moderate to severe dysplasia is often still surgical and aimed at eliminating the affected part of the transformation zone [2]. There are different type of surgical treatments (Large Loop Excision of the Transformation Zone (LLETZ), knife cone biopsy and laser conisation), the success rate is approximately 90% [3]. Historically, moderate and severe dysplasia was treated with cold knife biopsy. Nevertheless, with deep cones, hemorrhage, infection and post procedure stenosis were reported [4]. LLETZ seems to be a good alternative. This procedure could be performed under local anesthesia, is cheaper, less painful and seem to have less short and long term morbidity. Risk of residual disease is the same compared to cold knife cone [5]. Therefore, LLETZ is the golden standard for treating cervical dysplasia nowadays.

Still, there is uncertainty about the effects of LLETZ on short and long term in terms of recurrence, fertility and future pregnancy outcomes. Since women diagnosed with CIN are usually at their reproductive age, the effects on future fertility and pregnancy are of concern. Women with a shorter time interval from LLETZ to pregnancy seem to have an increased risk for spontaneous abortion [6]. Furthermore, a recent study described a higher subfertility rate in patients who underwent cervical surgery [7] Finally, several studies show a higher risk on preterm delivery and low birth weight [6, 8–11]. Systematic review and meta-analysis reported on LLETZ for CIN confirm an increased rate of preterm delivery (<32wks RR 1.98, 95% CI 1.31–2.98; <28wks RR 2.33, 95% CI 1.84–2.94), premature rupture of the membranes (RR 1.88, 95% CI 1.54–2.29) and low birth weight (< 2500 g RR 2.48, 95% CI 1.75–3.51) [8, 10]. One study reported a 10 fold higher risk for preterm deliveries after more than one conisation procedure in women with cervical dysplasia [12]. The risk of preterm birth could also be related to the volume of the cervical excision [13, 14]. However this research is performed in patients with one procedure, volume could be the same factor in patients with multiple procedures.

Apart from reproductive arguments there is an ongoing debate on the long-term outcome after treatment with surgical excision. Several studies show a recurrence rate of CIN 2–3 after treatment of 15–22% within 2 years [15]. Margin involvement seemed to be a risk factor for developing residual or recurrent cervical dysplasia [15]. Moreover, even after adequate treatment and follow up with normal smear results, patients who were treated for cervical intraepithelial dysplasia seem to have an excessive risk of cervical cancer compared to patients with normal primary smear test results [16, 17]. This risk is even almost 25 times higher in patients with abnormal smear test results than in patients with normal smear test results after treatment [18]. Recent studies found that most women with residual or recurrent disease test positive for HPV after treatment [19, 20]. Possibly this could be a tool for risk stratification in the follow up after treatment. Moreover, treatment failure could be related to the HPV status. This could raise the question if patients of this specific group could benefit from a non-invasive treatment modality to treat HPV and avoid further surgical treatment.

A potential agent in non-invasive therapy is imiquimod cream. Imiquimod 5% cream is a topical immune response modifier with indirect antiviral and antitumor properties. It is prescribed for HPV-associated genital warts, superficial basal cell carcinoma and actinic keratosis. Studies showed that it is a safe and effective treatment for usual type vulvar intraepithelial neoplasia (VIN), which is pathophysiologically comparable to CIN [21]. Furthermore, imiquimod seems also to be effective in primary CIN lesions [22]. It would also be helpful to determine a model to predict the responders to imiquimod therapy. This could select patients for non-ablative treatment.

The Topic 2 trial is a single blinded, randomized controlled trial with two intervention arms, in which imiquimod treatment is compared to standard treatment by LLETZ in patients with residual/recurrent CIN lesions after previous ablative treatment. The aim of the study is to investigate whether topical applied imiquimod is effective in the treatment of residual and recurrent CIN lesions.

## Methods

### Setting and study population

Patients are recruited at the moment in the Erasmus Medical Center, Rotterdam and in the Meander Hospital, Amersfoort, The Netherlands. This takes place at the outpatient clinic of gynecology. When non futility is proven after the pilot study, we intend to carry out a multi-center trial in the future throughout the Netherlands. Patients can be included if they have histologically confirmed residual or recurrent CIN lesions (CIN 1–3) after previous ablative treatment at least 6 months before the current diagnosis and have an age above 18 years. They are excluded if they have adenocarcinoma in situ, a history of (micro-) invasive cancer, hypersensitivity to the substance, immunodeficiency, pregnancy or lactation and insufficient knowledge of the English or Dutch language.

### Study objectives and outcome measures

The primary study objectives are:

1. To evaluate the effectivity of vaginal application of imiquimod 5% in the treatment of recurrent or persistent CIN. The primary outcome measure is reduction to normal cytology of the cervix at 26 weeks after start treatment in imiquimod and LLETZ group.

Secondary study objectives and outcome measures are:

1. To evaluate the effectivity of vaginal application of imiquimod 5% in the treatment of recurrent/residual CIN; reduction to absence of dysplasia in histology at 26 weeks as compared to baseline in the Imiquimod group.
2. Evaluate of the effect of treatment on HPV DNA positivity of CIN lesions, by comparison of PCR HPV-DNA detection at cervical biopsies/cytology taken at 0 and 26 weeks.
3. Establish the incidence and severity of side effects of LLETZ and imiquimod therapy.
4. Review the Quality of life (QoL) in patients at 0 and 20 weeks and after 1 year by the following QoL questionnaires: RAND 36, QLQ-C30 and QLQ-CX24.
5. Estimate the long term recurrence rate of CIN lesions measured by reduction to PAP1 by cytology examination at 6, 12 and 24 months after treatment. We will request the first outcome taken by the Dutch screening program for cervical cancer: cytology or HPV status will be obtained.

### Interventions

Patients will receive verbal and written information about the study procedure. They have to sign an informed consent, where after patients are randomized into the intervention arm or the standard treatment:

1. Intervention arm: Imiquimod treatment. Patients in this group have to insert imiquimod 5% cream intravaginally for 16 weeks.
2. Standard treatment. A LLETZ procedure is performed according to the current guidelines.

Patients in the imiquimod treatment group will apply imiquimod 5% cream intravaginally during 16 weeks. One sachet contains 12,5 mg of imiquimod and is applied with a vaginal applicator. The application frequency is 3 times a week. Patients get instructions and administer the cream themselves, before bedtime. The patients are advised to take an intravaginal shower with an applicator the next morning in order to remove cream leftovers, followed by an external shower to remove any remains on the vulva. Anti-inflammatory drugs (paracetamol or NSAID) can be used in case of mild systemic drug-related side effects. If the local or systemic side effects persist or are severe, patients are advised to reduce the frequency of the imiquimod insertion, first twice weekly, subsequently once weekly. Imiquimod treatment is discontinued for maximum of a week in case of persistent side effects. In order to prevent pregnancy, patients are advised to use adequate contraception. Subjects should refrain from vaginal sexual intercourse during the nights that imiquimod is applied until the vaginal shower the next morning. After 10 weeks a colposcopy is performed to rule out disease progression. Biopsies are only performed in case of suspicion of invasive disease.

In the standard treatment group, patients will undergo a LLETZ procedure within 4 weeks after the diagnosis. Excision of macroscopic lesions and the transformation zone will be achieved by a monopolar loop electrode, preferably under local anaesthesia.

Treatment efficacy is evaluated at 26 weeks follow-up for both groups. The Imiquimod group will have a Pap smear and a colposcopy with diagnostic biopsies. Biopsies are performed at the initial CIN lesion site and at any other suspect site, with a minimum of two. In case of persistent or progressive disease, surgical excision is performed. The LLETZ group will have follow-up with cervical smears at 6, 12 and 24 months according to the current guidelines.

This study protocol is almost identical to the TopIC-2 study, which is from the same study group. The TopIC-1 study studies the treatment of primary high grade CIN lesions with imiquimod or LLETZ procedure [22].

### Sample size calculation

The regression rate of a second LLETZ procedure is estimated 63% in a retrospective cohort (data not published). The regression rate of CIN lesions after treatment with imiquimod was based upon the only study reporting on the regression rate after 16 weeks of imiquimod therapy of primary CIN. This study showed a regression rate of 73% in patients with primary CIN lesions [23]. Since the effect could be lower in patients with residual or persistent CIN, we estimated the regression rate to be 60%.

To calculate the required sample size in this two-proportions non-inferiority trial we assume that the probability of regression is 63% for the standard (LLETZ procedure) while it is 60% after treatment with imiquimod.

Using a non-inferiority margin of 10% for the difference in proportions and a significance level (alpha) of 5%, a sample size of 174 per group is required to obtain a power of at least 80%. Allowing for 20% loss to follow-up the total required sample size is 433.

Because the uncertainty in the assumptions an interim analysis for futility will be performed by the Data Monitoring Committee (DMC) as soon as the primary outcome is available for 35 patients. This interim analysis is based on predictive power. That is the predicted probability of being able to prove non-inferiority given the results at the interim analysis. When the predictive power at this point is below 20% the trial will be stopped due to futility. Of course the DMC can always stop the trial due to safety concerns. The trial will never be stopped prematurely for efficacy so no alpha-adjustment is made.

### Randomization

Randomization is performed by use of a computerized randomization tool, to prevent selection and allocation bias.

### Blinding

The study is single blinded: the pathologists evaluating cytological and histological samples are blinded with respect to the intervention.

### Data collection

The coded data will be stored both on paper and in an electronic database. A digital case report form (e-CRF) is used. The data is accessible only to the principal and coordinating investigator. The following data are recorded:

#### Baseline (all patients)

- Patient characteristics: age, medical history, desire to have children, smoking, sexual behaviour.
- HPV genotype
- Quality of life for both treatment groups.

#### 6 weeks follow-up

- Adverse effects of imiquimod treatment: patient reported side effects and side effects noticed at clinical investigation.
- Adverse effects of LLETZ treatment: patient reported side effects.

#### 10 weeks follow-up

- Treatment compliance: amount of applied doses of imiquimod for the imiquimod group.
- Adverse effects of imiquimod treatment: patients reported side effects and side effects noticed at clinical investigation.
- Colposcopy with biopsies to determine progressive or invasive disease in the imiquimod group.

#### 16 weeks follow-up

- Adverse effects of imiquimod treatment: patient reported side effects and side effects noticed at clinical investigation.

#### 26 weeks follow-up

##### Imiquimod group

- Cervical cytology and HPV genotype.
- Quality of life
- Determination of grade of cervical dysplasia
- Treatment compliance: amount of applied doses of imiquimod.
- Adverse effects of imiquimod treatment: patients reported side effects and side effects noticed at clinical investigation.

##### LLETZ group

- Cervical cytology and HPV genotype.
- Quality of life
- With abnormal PAP smear, determination of grade of cervical dysplasia.

#### 12 and 24 months follow-up

- Cervical cytology outcomes for all treatment groups, including HPV genotyping.

### Statistical methods

Analysis will be done according to the intention to treat principle and the non-inferiority principle to show that imiquimod is not worse than an existing treatment (LLETZ). To investigate efficacy we will perform a per protocol analysis (although this is not the primary outcome of the study). The primary outcome is the difference between the probability of regression in the LLETZ and the imiquimod arms. The expected difference (LLETZ-imiquimod) will be calculated together with the 95% Aggresti confidence interval. If the upper bound of the interval lies below the non-inferiority margin of 10% non-inferiority of the imiquimod treatment is assumed to be proved. Logistic analysis of potential confounders (age at diagnosis, CIN grade, number of previous treatments, smoking, HPV-subtype) will be performed. Analysis will be based on intention to treat protocol.

The prevalence and severity of side effects of imiquimod and LLETZ treatment, as documented according to Common Terminology Criteria for Adverse Events guidelines, will be presented as proportions and means with 95% confidence intervals. Differences in the rates of overall side-effects and severe side-effects between the imiquimod and LLETZ groups will be tested with a chi-square test. Disease recurrence rates, defined by abnormal cervical cytology, after 6, 12 and 24 months will be evaluated in adequately treated patients by use of multiple logistic regression analysis, after adjustment for age at diagnosis, CIN grade, smoking sexual behavior and HPV subtype.

### Withdrawal of individual subjects and replacement

A study subject can stop the study at any time without explanation, this will have no consequences. The investigator can decide to withdraw a subject from the study for urgent medical reasons, non-compliance with the study procedures or pregnancy. There will be no replacement for withdrawn individuals. In principal a LLETZ will be performed, since this is still the standard treatment for patients with recurrent or persistent CIN lesions.

### Ethical considerations and dissemination

The standards outlined in the Declaration of Helsinki are guidelines for the study. Before the start of the study there was approval of the ethics committee. There is a data management safety board throughout the study. We will record adverse events and reported them to local protocol. We will be offering the study results for publication in international medical journals. If the subject agreed on this, study results will be communicated to trial participants by mail.

## Discussion

The development of a non-surgical treatment modality for residual and recurrent CIN lesions will lower the amount of LLETZ procedures for this indication and complications as a result of surgical intervention. Evidence shows that 15–22% of high-grade CIN lesions will persist or recur after 2 years and that patients after treatment for high grade CIN lesions will remain at higher risk for cervical cancer in the future. Based on earlier studies, we hypothesize that at least 50% of patients with high grade CIN will benefit from immunotherapy with imiquimod. The current study aims to test the treatment effectivity, while also assessing the clinical applicability of imiquimod treatment by documentation of side effects and quality of life associated with treatment.

## Abbreviations

CIN: Cervical Intraepithelial Neoplasia
HPV: Human Papillomavirus
LLETZ: Large Loop Excision of the Transformation Zone
VIN: Vulvar Intraepithelial Neoplasia
